# A Parallel-Group Randomised Waitlist-Controlled Trial of a Self-Guided Automated Online Psychoeducation Programme for Dementia Caregivers

**DOI:** 10.1192/j.eurpsy.2026.11584

**Published:** 2026-07-31

**Authors:** S.-T. Cheng

**Affiliations:** The Education University of Hong Kong, Tai Po, Hong Kong

## Abstract

**Introduction:**

Though psychoeducation with embedded psychotherapeutic components is beneficial for dementia caregivers, interventions tend to be regimented, without accommodating individual needs and preferences. As population ages and demand for accessible, personalised support grows, especially where in-person services are limited, the potential of web-based, self-guided programmes remains unclear.

**Objectives:**

Positive Dementia Caregiving in 30 Days (PDC30), the first fully automated, self-guided online intervention for dementia caregivers, was developed to be globally accessible and adaptable to diverse user needs. This study evaluated PDC30’s effectiveness in an international sample.

**Methods:**

Family dementia caregivers having been in the role for ≥3 months, providing ≥10 weekly care hours, scoring ≥5 on Patient Health Questionnaire-9 (PHQ-9), and passing a simple English comprehension test, were recruited via the internet. 274 eligible participants from 43 countries were allocated to treatment and 1-month waitlist-control using computer-generated simple randomisation. The intervention provided information on dementia care, interactive psychotherapeutic skill-building applications, and a generative artificial intelligence chatbot for personalised advice. Primary (depression) and secondary outcomes (anxiety, burden, and positive gains) were assessed via online questionnaires (PHQ-9, brief Profile of Mood States-Anxiety Subscale [POMS-A], brief Zarit Burden Interview [ZBI], and brief Positive Aspects of Caregiving Scale [PACS], respectively) at baseline and monthly intervals over three months. Intent-to-treat analysis was conducted using mixed-effects regression to examine trajectory changes in the outcome variables due to treatment. This completed trial was registered with ClinicalTrials.gov, NCT06409455.

**Results:**

At 1-month follow-up, coinciding with exclusive access to PDC30, intervention caregivers showed significant improvements in PHQ-9 (adjusted mean difference = ‑1.79, 95% CI = ‑2.93 to ‑0.64, p = 0.002, d = ‑0.37), ZBI (adjusted mean difference = ‑1.59, 95% CI = ‑2.69 to ‑0.49, p = 0.005, d = ‑0.34), and PACS (adjusted mean difference = 2.21, 95% CI = 1.33 to 3.10, p < 0.001, d = 0.59). Further bolstering the evidence, the differences generally disappeared after control participants gained access to the intervention, while improvements in both groups were sustained thereafter. No effect was observed for POMS-A. Participants reported using the website several times weekly, were generally satisfied with it, and found the chatbot most helpful.

**Conclusions:**

The findings demonstrate the feasibility, acceptability, and potential public health impact of PDC30, and support more developments of web-based, automated intervention for caregivers, especially in communities where conventional services are limited or inaccessible.

**Disclosure of Interest:**

None Declared

